# Augmentation Therapy With Serotonin 5‐HT_1A_ Receptor Partial Agonists on Cognitive Function in Depressive Disorders: A Systematic Review of Randomized Controlled Studies

**DOI:** 10.1002/npr2.70023

**Published:** 2025-05-27

**Authors:** Risa Yamada, Ayumu Wada, Andrew Stickley, Adrian Newman‐Tancredi, Tomiki Sumiyoshi

**Affiliations:** ^1^ Department of Preventive Intervention for Psychiatric Disorders National Institute of Mental Health, National Center of Neurology and Psychiatry Tokyo Japan; ^2^ Department of Psychiatry National Center Hospital of Neurology and Psychiatry Tokyo Japan; ^3^ Neurolixis Castres France

**Keywords:** 5‐HT_1A_ receptor biased agonists, 5‐HT_1A_ receptor partial agonists, azapirone derivative, cognitive impairment, depression

## Abstract

**Objective:**

The use of serotonin 5‐HT_1A_ receptor partial agonists (5‐HT_1A_‐PAs) as an add‐on therapy has been associated with the enhancement of attention/processing speed in patients with schizophrenia. Also, 5‐HT_1A_ receptors have been shown to play a role in the pathophysiology of mood disorders. There is compelling evidence supporting that stimulation of 5‐HT_1A_ receptors accelerates antidepressant effects. Accordingly, this systematic review examines the ability of adjunctive treatment with 5‐HT_1A_‐PAs to improve cognitive function in patients with depressive symptoms.

**Methods:**

A literature search using PubMed, the Cochrane Library, and Web of Science databases was performed from 1987 to January 2024 to identify randomized controlled trials (RCTs) corresponding to the following inclusion criteria: (1) RCTs, (2) human studies; studies that (3) targeted patients with a psychiatric disorder (except for schizophrenia or schizoaffective disorder), (4) evaluated the effect of cognitive functions, (5) were written in English.

**Results:**

From the 80 studies initially screened, three met the inclusion criteria. Two of these studies dealt with vascular depression while one focused on major depressive disorder (MDD). In MDD, combined treatment with buspirone and melatonin was more efficacious in ameliorating subjective cognitive disturbances compared to the use of buspirone alone or the use of a placebo. Likewise, the combination of escitalopram–tandospirone was more advantageous than escitalopram alone for improving executive function and verbal fluency in patients with vascular depression.

**Conclusions:**

Further studies with novel 5‐HT_1A_ receptor agonists are warranted to examine their potentially more robust benefits on cognitive performance in subjects suffering from mood deficits.

## Abstract 2

The adjunctive use of serotonin‐5‐HT1A partial agonists may be beneficial in improving cognitive function in patients with depressive symptoms. This systematic review of existing clinical trials provides support for this hypothesis, and indicate the need for further study.

## Graphical abstract

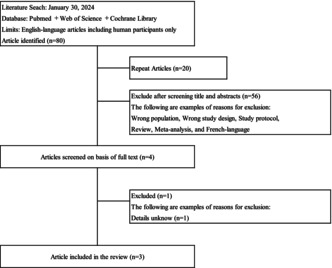

## 1 Introduction

Disturbances of cognitive function are common in psychiatric disorders, including schizophrenia and mood disorders [1, 2, 3]. Indeed, cognitive impairment in these psychiatric conditions may be associated with disturbances of several types of neural substrates involving cortical neurotransmissions, particularly an imbalance of glutamatergic signaling [4, 5].

Some patients with major depressive disorder (MDD) show impairment in memory, executive function, fluency, and attention/processing speed [3]. Specifically, persistent cognitive deficits in the remitted stage of MDD present a barrier to functional recovery in terms of occupational functioning and autonomy [6, 7]. Cognitive impairment is also common in bipolar disorder (BD) and occurs across several cognitive domains, as described above [1]. In fact, emerging evidence points to global cognitive deficits that are as severe as those observed in schizophrenia in 40%–50% of BD patients [8, 9]. Therefore, developing treatment approaches that target cognitive impairment is a priority for the betterment of psychiatric practice. This therapeutic strategy may be relevant to other psychiatric conditions characterized by mood symptoms, for example, vascular depression.

Previous reviews examined the effects of pharmacotherapy targeting serotonin 5‐HT_1A_ receptors on cognitive impairment in patients with schizophrenia [10, 11]. These receptors are located in key brain areas governing cognitive and emotional processes, for example, the frontal cortex, hippocampus, and amygdala [12]. The 5‐HT_1A_ receptor partial agonist (5‐HT_1A_‐PA) actions of atypical antipsychotic drugs, for example, aripiprazole, lurasidone, and brexpiprazole, preferentially increase extracellular concentrations of dopamine (DA) and acetylcholine in the prefrontal cortex relative to subcortical areas [13, 14, 15]. As enhancement of prefrontal DA transmissions is thought to regulate the activities of mesolimbic DA neurons [16, 17], 5‐HT_1A_ partial agonism may alleviate psychotic symptoms, in addition to cognitive impairment, in schizophrenia patients [11, 13]. At a clinical level, the authors' group was the first to report that add‐on use of the 5‐HT_1A_‐PAs buspirone or tandospirone is associated with cognitive enhancement in patients with schizophrenia [18, 19]. These pivotal studies prompted subsequent confirmation by independent groups of investigators, which overall indicate the benefits of the 5‐HT_1A_‐PA augmentation strategy for improving attention/processing speed, a key domain of cognition, as revealed by a meta‐analysis [11]. By contrast, a subsequent meta‐analysis did not show a significant effect of the adjunctive therapy on either verbal learning or working memory [10], suggesting that the cognitive benefits of 5‐HT_1A_‐PAs are domain‐specific.

There is compelling evidence that the 5‐HT_1A_ receptor plays a role in the pathophysiology of other psychiatric disorders, especially mood disorders. This is supported by the observation that ACTH release induced by the 5‐HT_1A_‐PA ipsapirone [20, 21, 22] is attenuated in depressed patients, indicating decreased sensitivity of the postsynaptic 5‐HT_1A_ receptor [23, 24, 25, 26]. In a positron emission tomography (PET) study, Drevets et al. [27, 28] showed that depressed patients with unipolar or bipolar disorder exhibited reduced 5‐HT_1A_ receptor binding potential in the medial temporal cortex and hippocampus, as well as in midbrain raphe, compared with healthy controls [27, 28]. Similarly, Sargent et al. [29] reported a widespread reduction (frontal, temporal, and limbic cortices) in 5‐HT_1A_ receptor binding in both medicated [29] and unmedicated [30] individuals with MDD, a finding which holds true for remitted patients [30]. A similar conclusion was reached by Hirvonen et al. [31], who reported reductions in receptor binding across large regions of the brain in drug‐naive individuals with MDD [31]. Accordingly, a postmortem study reported decreased 5‐HT_1A_ receptor numbers and receptor affinity in the hippocampi and amygdalae of depressed suicide victims, respectively [32]. A decreased 5‐HT_1A_ receptor binding was also found in the dorsolateral prefrontal cortex (DLPFC) in a small group of severely depressed patients [33], further supporting a role for 5‐HT_1A_ receptors in the pathophysiology of mood deficits.

Emerging evidence supports the role for agonist stimulation of 5‐HT_1A_ receptors as an adjuvant and accelerator of antidepressant effects [34, 35]. Consequently, several molecules with a 5‐HT_1A_ receptor partial agonist component, such as buspirone and related azapirones, have been investigated in preclinical studies and tested in clinical trials [10, 11, 18, 35]. The azapirone anxiolytic drugs buspirone and tandospirone, which function as 5‐HT_1A_‐PAs, are frequently used as adjunctive treatments for MDD patients who show an inadequate response to first‐line antidepressants [36]. For example, a meta‐analysis showed that buspirone and gepirone, both 5‐HT_1A_‐PAs, have some antidepressant activity [37]. However, these clinical benefits of 5‐HT_1A_‐PAs have not been sufficiently confirmed [37, 38], leading to the gradual deemphasis of this therapeutic avenue, although gepirone was eventually approved for the treatment of depression by the Food and Drug Administration [38].

As well as influencing mood, 5‐HT_1A_ receptors have been shown to play a key role in cognition and memory, leading to the interest in the use of 5‐HT_1A_ agonists to alleviate cognitive impairment, as shown in the animal models [35]. Regarding cognitive effects, most antidepressants (by themselves) only show small or negligible benefits in patients with MDD [39, 40]. This situation holds true also for BD in which various treatments (e.g., lithium, sodium, and antipsychotic drugs) have only yielded limited effects [41, 42]. However, there is little information on whether stimulation of the 5‐HT_1A_ receptor may improve cognitive function in patients with mood symptoms. This is a significant omission, as cognitive impairment has been shown to largely affect functional recovery in these patients, and comprehensive considerations on the potential ability of 5‐HT_1A_ (partial) agonists to alleviate cognitive deficits are lacking.

In this paper, we provide updated information on the potential role for several types of 5‐HT_1A_ agonists and related compounds in improving cognitive function in patients with mood deficits. Specifically, we conducted a systematic review of RCTs to examine the possible efficacy of 5‐HT_1A_‐PAs of the azapirone class for improving cognitive function in patients with depressive symptoms. We hypothesized that augmentation therapy with clinically available 5‐HT_1A_‐PAs, for example, buspirone and tandospirone, would be beneficial for improving specific domains of cognitive function in these patients.

## 2 Methods

### 2.1 Inclusion Criteria and Search Strategies

We conducted the current systematic review on the basis of the Preferred Reporting Items for Systematic Reviews and Meta‐Analyses (PRISMA) guidelines. The inclusion criteria were as follows: (1) RCTs, (2) human studies, (3) studies that targeted patients with a psychiatric disorder (*except for* schizophrenia or schizoaffective disorder), (4) evaluated the effect of cognitive functions, and (5) were written in English.

RY and AW independently conducted a literature search using PubMed, the Cochrane Library, and Web of Science databases from 1987 until 30 January 2024 using the following keywords: “alnespirone” OR “binospirone” OR “buspirone” OR “enilospirone” OR “eptapirone” OR “gepirone” OR “ipsapirone” OR “revospirone” OR “tandospirone” OR “zalospirone” AND “major depressive disorder” OR “major depression” OR “bipolar disorder” OR “mixed” OR “bipolar depression” OR “anxiety disorder” OR “generalized anxiety disorder” OR “social anxiety disorder” OR “panic disorder” OR “phobia” AND “cognition” OR “cognitive function.” Additional studies were obtained by scanning the reference lists of the included studies and previous reviews. TS approved the final list of included studies.

### 2.2 Data Extraction and Quality Assessment

The information for each study was independently extracted by RY and AW, with coding discrepancies resolved by TS. When the data were not fully described in the published article, the corresponding authors were contacted and asked to provide additional information. If there was no response to our queries, we tried to obtain the necessary information by measuring the length of graphs showing non‐tabulated results. If none of these methods proved feasible, then the studies were excluded from the analysis. Based on the information obtained, the outcome measures were classified into verbal learning, working memory, and executive function. The Cochrane risk of bias tool was used to evaluate the methodological quality of each RCT [43].

RY and AW independently assessed the following characteristics of each trial: (i) random sequence generation, (ii) blinding of subjects and personnel, (iii) blinding of outcome assessment, (iv) incomplete outcome data, (v) selective reporting, and (vi) other potential sources of bias. The assessment was done by evaluating what was reported in the selected articles and accessing and evaluating the study protocols where available. If necessary, any disagreements were thereafter resolved by TS.

## 3 Results

**FIGURE 1:**
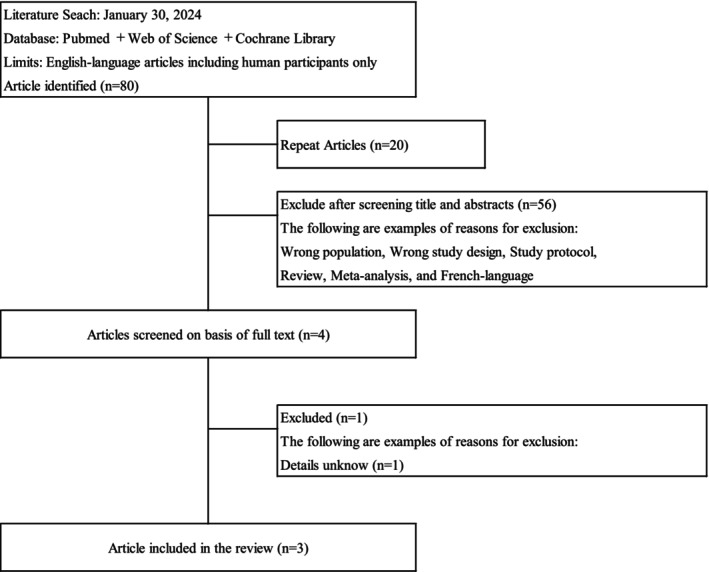
Preferred Reporting Items for Systematic Reviews and Meta‐Analyses (PRISMA) study selection flowchart.

Figure 1 shows the PRISMA study selection flowchart. The initial search yielded 80 potential articles. After removing duplicates, 60 articles were screened. The three studies included in the systematic review included 331 patients (190 men and 141 women).

### 3.1 Systematic Review

#### 3.1.1 Characteristics of Studies

Characteristics of the included studies are shown in Table 1. These studies were conducted in the United States [44] or China [45, 46], and included patients with MDD [44] or vascular depression [45, 46]. The mean age of the subjects in the former study was 42.4 years [44], while in the latter two studies [45, 46], the ages ranged from 69.0 to 70.9 years (in the control group) and from 68.0 years to 69.98 years (in the experimental group). The mean depressive symptoms severity score was 17.02, as measured by the patient‐rated Quick Inventory of Depressive Symptoms (QIDS‐SR), in the Targum et al.'s study [44], and ranged from 29.0 to 29.5 (in the experimental group), and from 28.0 to 29.0 (in the control group), as measured by the 17‐item Hamilton Depression Rating Scale, in the studies from China [45, 46]. The daily dose of 5‐HT_1A_‐PAs (buspirone or tandospirone) ranged from 10.0 to 15.0 mg/day, while the mean duration of its use ranged from 6 to 8 weeks. Escitalopram was used as a concomitant SSRI in two studies [45, 46]. The Targum et al.'s [44] study used three groups: augmentation with melatonin in a combination group, a buspirone only group, and a placebo group.

**TABLE 1:** Characteristics of the included studies.

	Study	Diagnosis	Sample size	Male/female	Treatment	Age, year (SD)	Symptom severity scores	Duration (week)	Dose of Bus or Tan (mg)	Assessment tools	Results
1	Targum et al. [44]	Major depressive disorder	134	87/47			QIDS‐SR (SD)			CGI, QIDS‐SR	Significant effects in
					Bus + MLA (3 mg)	43.1 (12.06)	16.98 (3.079)	6	15	IDS‐C, HAMA	MGH‐CPFQ (combination > others),
					Bus	40.8 (12.55)	16.88 (2.306)			MGH‐CPFQ	
					Pbo	42.7 (11.62)	17.24 (3.307)				
2	Chen et al. [45]	Vascular depression	89	47/42			HAMD (SD)			HAMD, HAMA, MMSE	Significant effects in MMSE
					Tan + ESCIT (10 mg)	69.98 (4.44)	29.45 (2.53)	8	10	Fazekas scale score	Significant time effect
					ESCIT (10 mg)	70.93 (4.47)	28.98 (2.3)			NIHSS, CGI	
3	Chen et al. [46]	Vascular depression	108	56/52			HAMD (IQR)				
					Tan+ESCIT (10 mg)	68.0	29.0 (26.0, 31.0)	8	10	HAMD, HAMA, RAVLT	Significant effects in SVF and TMT
					ESCIT (10 mg)	69.0	28.0 (27.0, 31.0)			SVF, TMT, DST, CDT	Significant time effect

Abbreviations: Bsu, Buspirone; CDT, Clock Drawing Test; CGI, Clinical Global Impression Scale; CPFQ, Cognitive and Physical Functioning Questionnaire; DST, Digital Span Test; ESCIT, Escitalopram; HAMA, Hamilton Anxiety Rating Scale; HAMD, Hamilton Depression Rating Scale; IDCS‐C, Inventory of Depressive Symptomatology—Clinical version; IQR, interquartile range; MGH, Massachusetts General Hospital; MMSE, Mini‐Mental State Scale; MLA, melatonin; NIHSS, National Institutes of Health Stroke Scale; QIDS, Quick Inventory of Depressive Symptoms; RAVLT, Rey Auditory Verbal Learning Test; SD, Standard Deviation; SR, Self Report; SVF, Semantic Verbal Fluency; Tan, Tandospirone; TMT, Trail Making Test.

There were considerable differences between the studies in terms of the cognitive outcome measures used. Thus, the Targum et al.'s study used the Massachusetts General Hospital Cognitive and Physical Functioning Questionnaire (MGH‐CPFQ) [47], which is a validated patient‐rated instrument that evaluates distinct physical and cognitive symptoms [44]. On the other hand, Chen et al. [45] used the Mini‐Mental State Scale (MMSE) [48] to detect cognitive impairments [45]. In addition, the same authors [46] also used the Rey Auditory Verbal Learning Test (RAVLT), Semantic Verbal Fluency (SVF), Trail Making Test (TMT), Digital Span Test (DST), and Clock Drawing Test (CDT) [46].

#### 3.1.2 Cognitive Function

Cognitive benefits, including those for executive problems, general cognitive disturbances, and difficulties of memory and spatial learning, were evident (Tables 2, 3, 4). In the case of patients with MDD [44], there was a significant effect of the medication on cognitive function in the combination treatment group (buspirone plus melatonin) when compared to the pooled buspirone and placebo group (Table 3). Regarding patients with vascular depression, escitalopram augmentation with tandospirone resulted in significant improvements in various cognitive domains, such as executive function (TMT) and verbal fluency (SVF) [45, 46] (Table 4).

**TABLE 2:** Neurocognitive measures used in the reviewed studies.

	Study	Evaluation type	Measures	Cognitive domains
1	Targum et al. [44]	Subjective	MGH‐CPFQ	
			Physical items	Sleepiness and fatigue
			Cognitive items	Apathy, inattention, forgetfulness, word‐finding difficulties, mental slowness
2	Chen et al. [45]	Objective	MMSE	General cognition (orientation, memory recall, working memory, concentration, language, and visuospatial)
3	Chen et al. [46]	Objective	RAVLT	Verbal memory
			SVF	Verbal fluency
			TMT	Visual scanning, graphomotor speed, and executive function
			DST	Attention, and processing speed
			CDT	Executive functioning, visuospatial skills, working memory, attention, language skills, numerical ability

Abbreviations: CDT, Clock Drawing Test; DST, Digital Span Test; MMSE, Mini‐Mental State Examination; RAVLT, Rey Auditory Verbal Learning Test; SVF, Semantic Verbal Fluency; TMT, Trail Making Test.

**TABLE 3:** Effect of buspirone on cognition performance in major depressive disorder.

Study	Measures	Buspirone + melatonin		Buspirone		Placebo	
		Baseline mean score	Posttreatment mean score	Baseline mean score	Posttreatment mean score	Baseline mean score	Posttreatment mean score
Targum et al. [44]	Total MGH‐CPFQ	27.8	21.31	27.52	24.21	27.53	22.37
	All cognitive items	19.61	14.81	19.38	17.17	19.33	15.87

*Note:* Cognitive items include apathy, inattention, forgetfulness, word‐finding difficulties, and mental slowness.

Abbreviations: CPFQ, Cognitive and Physical Functioning Questionnaire; MGH, Massachusetts General Hospital.

**TABLE 4:** Effect of tandospirone on cognition performance in vascular depression.

Study	Scles	Tandospirone add‐on		Control		*p*
		Baseline mean score	Posttreatment mean score	Baseline mean score	Posttreatment meanscore	
Chen et al. [45]	MMSE	20.75	26.45	20.62	20.96	< 0.001
Chen et al. [46]	RAVLT	33.0	33.0	32.0	32.0	0.989
	SVF	13.0	14.0	13.0	13.0	< 0.001
	TMT	115.0	108.0	119.0	117.0	< 0.01
	DST	6.0	6.0	6.0	6.0	0.424
	CDT	4.0	4.0	4.0	4.0	0.777

Abbreviations: CDT, Clock Drawing Test; DST, Digital Span Test; MMSE, Mini‐Mental State Scale; RAVLT, Rey Auditory Verbal Learning Test; SVF, Semantic Verbal Fluency; TMT, Trail Making Test.

#### 3.1.3 Risk of Bias

The summary for the risk of bias is shown in Table 5. All three studies had a noticeable rating in relation to either the blinding of the participants and personnel, blinding of outcome assessments, and/or selective reporting. Regarding other sources of bias, the risk was unclear in all of the studies.

**TABLE 5:** Risk of bias.

	Random sequence generation	Allocation concealment	Blinding of participants and personnel	Blinding of outcome assessment	Incomplete outcome data	Selective reporting	Other sources of bias	Overall
Targum et al. [44]	Low	Low	Low	Low	Low	High	Unclear	High
Chen et al. [45]	Low	Low	High	Some concern	Low	Low	Unclear	High
Chen et al. [46]	Low	Low	High	Some concern	Low	Unclear	Unclear	High

## 4 Discussion

To our knowledge, this is the first systematic review regarding the benefits of the adjunctive use of 5‐HT_1A_‐PAs (buspirone and tandospirone) for potentiating the treatment of cognitive impairment in patients characterized by depressive symptoms. Until now, these marketed 5‐HT_1A_‐PAs have been extensively reported to alleviate only anxiety [49] and depressive symptoms [50].

The studies examined here focused on patients with MDD [44] or vascular depression [45, 46]. In the context of MDD, Targum et al. [44] demonstrated that improvement of subjective cognition, as assessed by the Cognitive and Physical Functioning Questionnaire (CPFQ), was most pronounced in patients receiving a combination of buspirone (15 mg) and melatonin‐SR (3 mg) compared to those receiving buspirone alone or placebo. For vascular depression, Chen et al. [45] reported that objective cognitive performance, measured using the Mini‐Mental State Examination (MMSE), improved significantly more in the tandospirone + escitalopram group than in the escitalopram alone group. Furthermore, Chen et al. [46] found that tandospirone demonstrated superior efficacy compared to placebo in enhancing objective cognitive performance, as measured by the Semantic Verbal Fluency (SVF) test and the Trail Making Test (TMT, B‐A), which assess verbal fluency and working memory, respectively, over the observation period.

Collectively, these findings suggest that in patients with depressive disorders, the adjunctive administration of buspirone or tandospirone may be effective in enhancing both subjective and objective cognitive domains governed by the prefrontal cortex. Accordingly, an RCT conducted by Wang et al. [51] evaluated the long‐term effects (24 weeks) of buspirone on attention and processing speed in patients with schizophrenia. Similar prolonged effects of 5‐HT_1A_‐PAs on cognitive function would be expected in patients with mood disorders.

Several mechanisms may underlie the ability of 5‐HT_1A_ ‐PAs, such as the above azapirone derivatives, to improve cognition. These include (1) enhancement of dopaminergic and cholinergic neurotransmission in the prefrontal cortex and/or hippocampus [52, 53], (2) preferential activation of cortical 5‐HT_1A_ receptors, with negative feedback on 5‐HT release via presynaptic 5‐HT_1A_ autoreceptors [54, 55], (3) promotion of neuroplasticity of hippocampal neurons [56], and (4) neuroprotective actions against pathological processes causing cognitive impairment in vascular depression [57, 58, 59]. It is also possible that non‐serotonergic mechanisms may be involved in the actions of buspirone and tandospirone, for example, antagonism at alpha2‐adrenoceptors by 1‐PP, the principal metabolite of the azapirone 5‐HT_1A_‐PAs [60, 61]. Further investigation is therefore needed to clarify the potential cognitive benefits of novel compounds to be developed based on the above considerations.

The mechanisms underlying the efficacy of 5‐HT_1A_ ‐PAs in treating depressive symptoms and cognitive function in mood disorders may involve several neural substrates. For example, improvement in depressive symptoms may be primarily mediated through 5‐HT_1A_ receptor‐induced neuroplasticity and serotonin release regulation [34]. In contrast, the mechanisms underlying improvement in cognitive function may involve activation of 5‐HT_1A_ receptors in the cortex and hippocampus, as well as enhancement of adult neurogenesis [62]. These considerations may help explain the possible difference in the degree of improvement between cognitive deficits and depressive symptoms in patients who receive augmentation therapy with 5‐HT_1A_ ‐PAs [35].

The clinical benefits of 5‐HT_1A_ receptor activation have also been reported for compounds other than azapirones. For example, the efficacy of the adjunctive use of brexpiprazole, an antipsychotic drug with a potent affinity and partial agonist activity at 5‐HT_1A_ receptors [63], may be applicable to cognitive impairment in schizophrenia, MDD, and bipolar depression [64, 65, 66]. So far, in MDD patients with anxiety symptoms, adjunctive treatment with the drug has been found to improve functional ability, such as the relation to social and family life, in addition to reducing core depressive symptoms [66]. Further, the use of brexpiprazole was found to improve quality of life in patients with bipolar depression [65].

As for the development of novel 5‐HT_1A_ agonists, NLX‐101 (a.k.a. F15599), a *biased* agonist selectively acting on postsynaptic cortical neurons [54, 67, 68, 69], is attracting increasing attention. For example, the drug has been reported to alleviate working memory in a variety of animal models [54, 67, 68, 69]. These findings may encourage clinical trials of novel 5‐HT_1A_ receptor‐related compounds for cognitive enhancement in patients with various psychiatric disorders. In this context, the recent re‐emergence of enthusiasm for serotonergic psychedelics may provide useful information on the role of 5‐HT in cognitive deficits in depression [70, 71]. Indeed, psychedelics act as agonists at multiple 5‐HT receptors, including 5‐HT_1A_ receptors [72], so they may provide some cognitive benefits in subjects with depressive symptoms via activation of these receptors.

So far, some information has been obtained on neuroimaging data to elucidate the mechanism underlying the efficacy of 5‐HT_1A_ ‐PAs in improving cognitive function in individuals with depressive symptoms. For instance, NLX‐112 (befiradol, F13640) is among the most extensively studied compounds in this context. In microdosing PET imaging, this 5‐HT_1A_ agonist was radiolabeled, resulting in the generation of 18F‐F13640 (18F‐NLX‐112)—the first full agonist PET radiopharmaceutical targeting 5‐HT_1A_ receptors [73]. By visualizing functionally active 5‐HT_1A_ receptors, the radiolabeled compound has provided critical insights into its cerebral bioavailability, distribution, and pharmacokinetics in both animal models and human studies [73, 74, 75]. Further research integrating advanced neuroimaging methodologies, such as PET and fMRI, may offer a deeper understanding of how 5‐HT_1A_ receptor‐targeted pharmacotherapy modulates cognitive function, for example, through dopaminergic, glutamatergic, and/or cholinergic systems.

Limitations of the present systematic review should be stated. First, caution should be exercised regarding the generalizability of the present findings given the small number of included studies, which might have been influenced by selective reporting of positive results. Second, all of the examined studies focused on relatively short‐term (6–8 weeks) outcomes. Further research on the longer term benefits of 5‐HT_1A_‐PAs is needed to confirm the findings, as discussed above. Third, two of the three studies examined here were conducted in single‐center settings with a small sample size, so caution should be exercised before generalizing the present findings to other populations. Fourth, there is a variance in the type of cognitive tests used in the studies analyzed in this review. The use of uniform cognitive tests or batteries should have provided more precise information on whether or not 5‐HT_1A_ agonists would improve specific or entire domains of cognitive function. These considerations overall indicate the need for further clinical trials on cognitive benefits of various types of 5‐HT_1A_ agonists.

## 5 Conclusions

A small number of studies have been carried out suggesting that the adjunctive use of some 5‐HT_1A_‐PAs may be advantageous in improving cognitive function in patients with depressive symptoms. Although the available data is limited, it provides support for undertaking further research to examine the cognitive benefits of 5‐HT_1A_ receptor activation. Moreover, novel compounds displaying selective or biased agonist properties at 5‐HT_1A_ receptors could exhibit even better therapeutic activity and increase the chance of functional recovery in patients suffering from depressive symptoms.

## Author Contributions

R.Y. reviewed the literature and wrote the first and subsequent drafts of the manuscript. T.S. developed the study concept and hypothesis. R.Y., A.W., and T.S. managed the data collection and contributed to the interpretation of the results. T.S. and A.S. assisted in writing the manuscript and critically reviewed the drafts. T.S. and A.N.‐T. contributed to the interpretation of the results, revised drafts of the manuscript, and provided feedback with particular expertise. All authors contributed to and have approved the final version of the manuscript.

## Ethics Statement

The authors have nothing to report.

## Consent

The authors have nothing to report.

## Conflicts of Interest

Dr. Sumiyoshi is an Editorial Board member of Neuropsychopharmacology Reports and a co‐author of this article. To minimize bias, they were excluded from all editorial decision‐making related to the acceptance of this article for publication.

## Data Availability

Data sharing not applicable to this article as no datasets were generated during the current study.
